# Lessons learned from a case of placenta previa totalis complicated by placenta accreta spectrum: Importance of timely decision-making

**DOI:** 10.1016/j.ijscr.2024.110804

**Published:** 2024-12-29

**Authors:** Ibrahim Fathallah, Ayham Qatza, Abd Alrhman Alajrd, Majd Al-Ali

**Affiliations:** a Faculty of Medicine, Al-Baath University, Homs, Syria; b Faculty of Medicine, Hama University, Hama, Syria; c Department of Obstetrics and Gynecology, Tishreen University, Lattakia, Syria

**Keywords:** Placenta previa, Placenta percreta, Placenta accreta spectrum, Case report

## Abstract

**Introduction and clinical importance:**

Placenta previa (PP) is characterized by abnormal placental placement in the lower uterine segment, obstructing the cervical opening. Placenta previa totalis (PPT) occurs when the placenta completely covers the internal cervical os. This condition can lead to placenta accreta spectrum (PAS), where the placenta adheres abnormally to the uterine wall, complicating separation. PAS is reported in approximately 0.2 % of pregnancies.

**Case presentation:**

This report concerns a case of a 28-year-old woman at 35 weeks gestation presented with painless vaginal bleeding and contractions. She had a history of five cesarean sections. Ultrasound revealed PPT with suspected placenta percreta, alongside multiple false knots and blood lakes in the placenta. A vertical uterine incision allowed for the delivery of a 2700-g male fetus, with APGAR scores of 9/10 and 10/10. Following massive hemorrhage, an abdominal hysterectomy was performed. The patient was discharged on postoperative day four in stable condition, and the infant was also discharged healthy.

**Clinical discussion:**

PAS poses significant maternal risks, necessitating early diagnosis and meticulous surgical planning. Management strategies, including feeder vessel ligation and conservative approaches, aim to minimize hemorrhage. Hysterectomy remains a critical intervention in cases of uncontrollable bleeding, with postoperative care focused on hemodynamic stabilization and pain management.

**Conclusion:**

This paper emphasizes effective communication, timely decision-making, and adherence to massive hemorrhage protocols in managing obstetric emergencies like PAS. In addition, routine screening during second-trimester ultrasounds for high-risk pregnancies and further prospective clinical trials are needed to enhance diagnostic and management strategies.

## Highlights

• Placenta previa totalis (PPT) can lead to placenta accreta spectrum (PAS), increasing hemorrhage risk during separation.
• PAS is reported in approximately 0.2% of pregnancies, but the risk escalates significantly with multiple cesarean deliveries.
• Ultrasound is essential for diagnosing PPT and evaluating complications such as placenta percreta and blood lakes.
• Planned cesarean hysterectomy is standard for high-risk or confirmed PAS to reduce severe hemorrhage.
• Post-surgery, close monitoring for hemorrhage and complications is crucial for maternal and neonatal safety.

## List of abbreviations

PP: Placenta previa
PPT: Placenta previa totalis
PAS: Placenta accreta spectrum
FIGO: The Federation International Federation of Gynaecology and Obstetrics
DIC: Disseminated intravascular coagulation
MRI: magnetic resonance imaging
ICU: Intensive care unit

## 1 Introduction

Placenta previa (PP) is termed abnormal placental placement in the lower uterine segment, obstructing the cervical opening either partially or fully. The condition in which the placenta completely covers the internal cervical os is defined as placenta previa totalis (PPT). It can progress to a more severe condition known as placenta accreta spectrum (PAS), where the placenta abnormally adheres to the uterine wall, making separation impossible [1]. For women diagnosed with PP, the risk of placenta accreta is 3 % following the first cesarean and rises to 67 % after five or more cesareans [2]. The combination of PPT and PAS can result in severe bleeding during pregnancy and childbirth. Furthermore, it significantly increases the likelihood of adverse outcomes for both the mother and the fetus, including illness and death [3]. Among all types of placenta accreta, placenta percreta is associated with the greatest risk of severe bleeding before, during, and after childbirth [4]. Eventually, for patients with a high risk or confirmed diagnosis of PAS, planned cesarean hysterectomy is the current standard treatment [5]. This paper describes the first case of PPT complicated with PAS from Syria and provides a detailed overview of the patient's condition, diagnostic approach used, treatment strategy, and outcomes, aiming to increase awareness of this rare case.

This work is also reported in line with SCARE criteria, which helped to improve the transparency and quality of this case report [6].

## 2 Case presentation

**Fig. 1:**
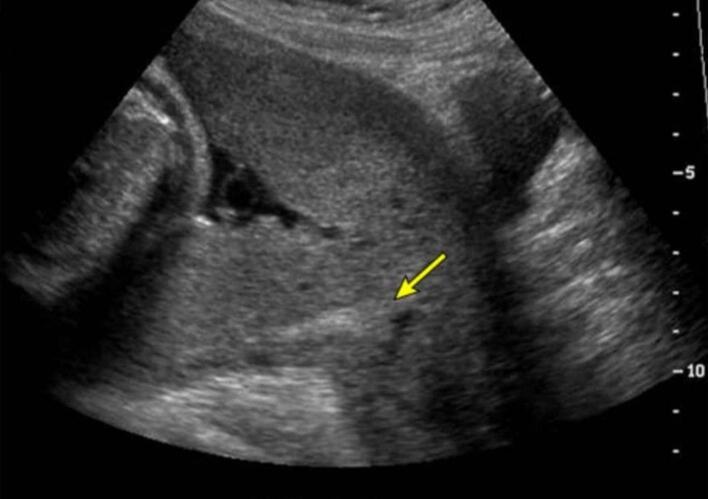
An abdominal ultrasound findings were as follows: several blood lakes within the placenta and a placenta previa totalis, which invaded the uterine wall.

**Fig. 2:**
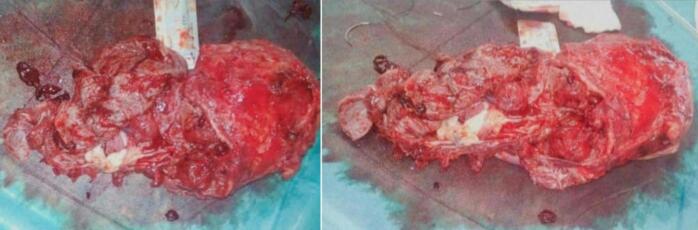
Gross picture of placenta invading myometrium after hysterectomy.

A 28-year-old pregnant woman at 35 weeks and 1 day of gestation presented to the obstetrics and gynecology department with painless vaginal bleeding and uterine contractions of 15 min duration. She denied hematuria but reported dark-colored urine. The patient had no unusual symptoms or signs during the whole pregnancy. In addition, she was pregnant for the sixth time, with no complications in the recent or previous pregnancies. She underwent five previous cesarean sections. The medical, allergic, genetic, and psychosocial histories were normal, and she denied the use of tobacco, alcohol, and drugs. Vital signs on admission were: blood pressure, 70/100 mmHg; pulse, 88 beats/min; tympanic temperature, 37.5C; and oxygen saturation, 96 % on room air. An abdominal ultrasound finding was as follows: a single live fetus at 35 weeks and 1 day in the breech position and a PPT, suggesting a centrally located placenta that completely covers the internal os of the cervix, invaded the uterine wall. Furthermore, multiple false knots in the umbilical cord and several blood lakes within the placenta were observed [Fig. 1]. Fetal heart rate was reactive (positive), and amniotic fluid volume was adequate. Differential diagnosis included placental abruption, but a transabdominal ultrasound confirmed the diagnosis of PPT with suspected placenta percreta. She was admitted to the operating room for an emergency cesarean section with a total abdominal hysterectomy in the context of vaginal bleeding, and previous cesareans. Laboratory results showed a hemoglobin of 5 g/dL and a platelet count of 250,000/μL. Therefore, four units of blood were transfused preoperatively, raising the hemoglobin to 8.5 g/dL. Prophylactic antibiotics were made with 1500 mg of rosactam. The surgical procedure was initiated, with a vertical incision in the uterine fundus and the extraction of the male fetus weighing 2700 g, where the APGAR (Appearance, Pulse, Grimace, Activity, Respiration) scores were 9/10 in the first minute and 10/10 in the fifth minute for live birth. Subsequently, placenta percreta with massive hemorrhage was identified, leading to the decision for a life-saving abdominal hysterectomy [Fig. 2]. The massive hemorrhage protocol was activated, and the following blood products were administered: a total of 20 units of blood, 6 units of plasma, 2 units of platelets, and 3 units of albumin. Electrolyte imbalances, specifically hypocalcaemia, were corrected with 2500 mg of calcium gluconate. Postoperatively, the patient underwent postoperative care protocols, which indicated hemodynamic stability; however, her bladder was displaced. Although the placenta did not invade the bladder, the ureters were dilated and remained so for 4 days. Two ureteral stents were placed. The patient was discharged on the fourth postoperative day in good general condition with normal bowel and bladder function. The stents were removed, and the infant was also discharged in good health.

## 3 Discussion

Placenta percreta is a severe obstetric complication characterized by placental villi penetrating the myometrium into the uterine serosa and possibly adjacent organs. It is one of three degrees of morbidly adherent placenta: placenta accreta, increta, and percreta, accounting for 7 % of cases [7]. A new classification system for PAS has been proposed by the Federation International Federation of Gynecology and Obstetrics (FIGO), replacing earlier categorical terminology (placenta accreta, increta, and percreta) [8]. Based on reported studies, PAS occurs in approximately 0.2 % of all pregnancies [9]. The most important risk factors for PAS include a history of surgical procedures or endometrial manipulation and the presence of PP following cesarean section [2]. In addition, advanced maternal age, repeated births, and a history of dilation and curettage are well-established risk factors for abnormal placentation [10]. Given our patient's significant risk factors, timely surgical planning is essential. Complications of PAS vary from localized destruction of the uterine wall to severe bleeding, end-organ damage, disseminated intravascular coagulation (DIC), bladder perforation, fistula formation, ileus, and lactation failure [11]. Therefore, early diagnosis of invasive placenta is crucial for developing a comprehensive management plan to ensure maternal safety and minimize postpartum hemorrhage. For instance, severe bleeding leading to hemorrhagic shock or DIC during childbirth significantly increases maternal morbidity and mortality. These complications are clinically associated with all three types of abnormal placentation. To confirm the diagnosis of abnormal placentation, preoperative color Doppler imaging and ultrasound and magnetic resonance imaging (MRI) may yield more definitive results [10]. Regarding hemorrhage control, prior research has demonstrated that surgical ligation of feeder vessels can decrease the risk of substantial postpartum hemorrhage and may obviate the need for hysterectomy. This technique is considered an effective procedure in the management of placenta accreta [12]. Another study reported that partial placental removal combined with conservative surgery resulted in less operative time and blood loss compared to radical hysterectomy. However, for severe shapes of PAS, the outcomes were similar [13]. Given the heightened risk of significant hemorrhage, either immediate or delayed, associated with fertility preservation and conservative management of adherent placenta, hysterectomy may become the optimal course of action [14]. Furthermore, advances in managing PAS include interventional radiology approaches, such as preoperative arterial catheterization, aimed at occluding pelvic blood flow to reduce intraoperative hemorrhage. More research is necessary to confirm the safety and effectiveness of these techniques and develop standardized protocols [15]. The presence of uncontrollable bleeding necessitated the performance of a hysterectomy in this instance. Early postoperative care involves diligent monitoring, often best performed in the intensive care unit (ICU), depending on the need for organ support or transfusions to ensure hemodynamic and haemorrhagic stabilization. In addition, a multimodal approach for pain management should be offered, including paracetamol, NSAIDs, neuraxial analgesia, and/or truncal blocks like the transversus abdominis plane block [2]. In this case, the patient showed rapid recovery in the PACU, allowing for sufficient monitoring and care.

## 4 Conclusion

This case underscores the necessity of effective communication among medical team members, timely decision-making, and adherence to massive hemorrhage protocols in managing obstetric emergencies like PAS. In addition, it is important that clinicians screen all pregnancies during routine second-trimester ultrasounds for these conditions, especially for high-risk patients like those with multiple prior cesarean deliveries. Significant knowledge gaps exist in diagnosing and managing placenta previa and PAS, with recommendations largely based on expert opinion rather than quality evidence. Prospective clinical trials are needed to improve outcomes.

## CRediT authorship contribution statement

Ibrahim Fathallah: Writing – review & editing, Writing – original draft, Data curation.

Ayham Qatza: Writing – review & editing, Writing – original draft.

Abd Alrhman Alajrd: Writing – review & editing, Writing – original draft.

Majd Al-Ali: Writing – review & editing, Supervisor.

Ayham Qatza: submitted the final manuscript.

All authors read and approved the final manuscript.

## Consent

Written informed consent was obtained from the patient for publication of this case report and any accompanying images. A copy of the written consent is available for review by the Editor-in-Chief of this journal on request.

## Ethics approval and consent to participate

Ethics clearance was not necessary since the University waives ethics approval for publication of case reports involving no patients' images, and the case report is not containing any personal information. The ethical approval is obligatory for research that involve human or animal experiments.

## Funding

The author(s) received no financial support for the research, authorship, and/or publication of this article.

## Declaration of Generative AI and AI- assisted technologies in the writing process

None.

## Declaration of competing interest

The author(s) declared no potential conflicts of interest with respect to the research, authorship, and/or publication of this article.

## Data Availability

Data sharing not applicable to this article as no datasets were generated or analyzed during the current study.
